# The first human heart transplant and further advances in cardiac transplantation at Groote Schuur Hospital and the University of Cape Town

**Published:** 2009-02

**Authors:** Johan G Brink, Joannis Hassoulas

**Affiliations:** Chris Barnard Division of Cardiothoracic Surgery, University of Cape Town, Groote Schuur and Associated Academic Hospitals, Cape Town; Department of Cardiac Surgery, Medical School, University of Crete, Heraklion, Crete, Greece

## Abstract

**Summary:**

Christiaan (Chris) Barnard was born in 1922 and qualified in medicine at the University of Cape Town in 1946. Following surgical training in South Africa and the USA, Barnard established a successful open-heart surgery programme at Groote Schuur Hospital and the University of Cape Town in 1958. In 1967, he led the team that performed the world’s first human-to-human heart transplant. The article describing this remarkable achievement was published in the *South African Medical Journal* just three weeks after the event and is one of the most cited articles in the cardiovascular field. In the lay media as well, this first transplant remains the most publicised event in world medical history.

Although the first heart transplant patient survived only 18 days, four of Groote Schuur Hospital’s first 10 patients survived for more than one year, two living for 13 and 23 years, respectively. This relative success amid many failures worldwide did much to generate guarded optimism that heart transplantation would eventually become a viable therapeutic option.

This first heart transplant and subsequent ongoing research in cardiac transplantation at the University of Cape Town and in a few other dedicated centres over the subsequent 15 years laid the foundation for heart transplantation to become a well-established form of therapy for end-stage cardiac disease. During this period from 1968 to 1983, Chris Barnard and his team continued to make major contributions to organ transplantation, notably the development of the heterotopic (‘piggy-back’) heart transplants; advancing the concept of brain death, organ donation and other related ethical issues; better preservation and protection of the donor heart (including hypothermic perfusion storage of the heart; studies on the haemodynamic and metabolic effects of brain death; and even early attempts at xenotransplantation.

## Summary

Christiaan Barnard with his team, performed the world’s first human-to-human heart transplant operation on 3 December 1967. It was a major historical event and a significant breakthrough for medical science. The article describing this remarkable achievement, titled ‘A human cardiac transplant: an interim report of a successful operation performed at Groote Schuur Hospital, Cape Town’ was published just three weeks after the event in a special edition of the South African Medical Journal.^1^ This must rank as one of the most rapidly published medical reports of all time.

**Fig. 1.**
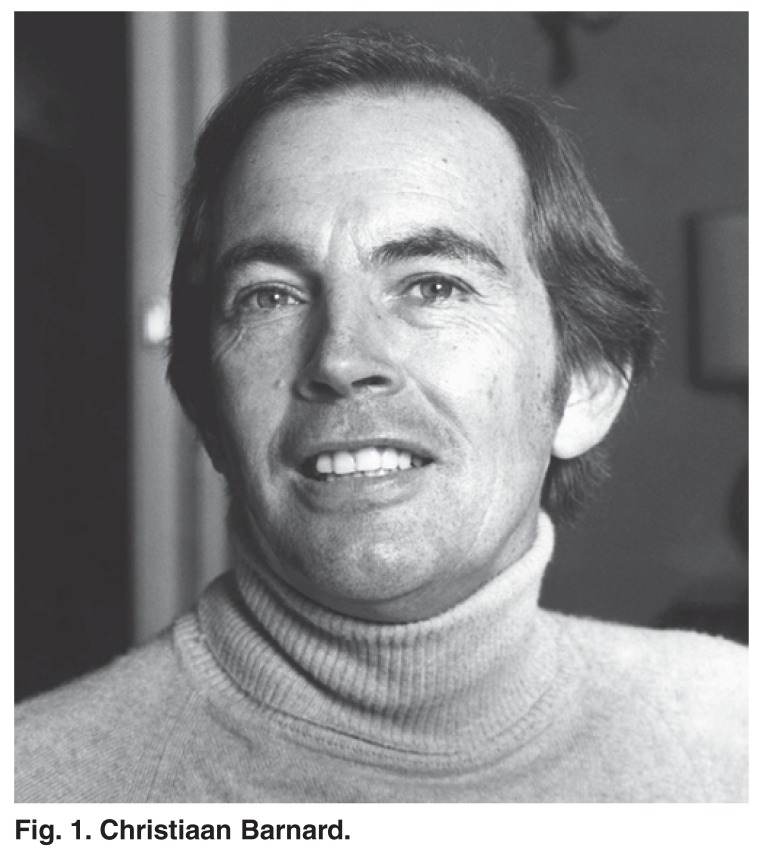
Christiaan Barnard.

**Fig. 2.**
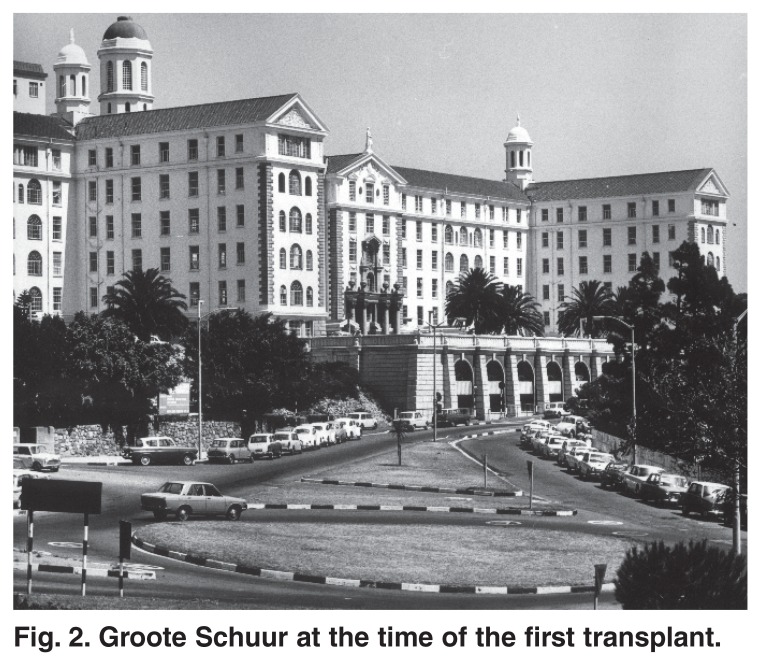
Groote Schuur at the time of the first transplant.

Media coverage around the world of this event and subsequent transplants was front page and appeared daily for weeks and months on end, describing all aspects in detail and giving progress reports on the postoperative course of the patients. This degree of public acclaim had not been previously experienced by any other physician or surgeon and nor would it be experienced subsequently. This was in part because the dramatic nature of the operation had captured the public’s imagination, but was equally a response to Chris Barnard’s youthful good looks and charismatic personality (Fig. 1). Barnard’s name and that of the University of Cape Town and Groote Schuur Hospital (Fig. 2) are inextricably associated with the first heart transplant

## The first transplant

On the night of 2/3 December 1967, Barnard performed the world’s first human-to-human orthotopic heart transplant in his patient, Louis Washkansky. Today, when heart transplantation has become a relatively routine and commonplace procedure, one may be inclined to underestimate Barnard’s immense courage in undertaking this first operation. Washkansky, a 53-year-old man with severe coronary insufficiency, was far from an ideal recipient by today’s standards, being a diabetic and a smoker with peripheral vascular disease. Furthermore, his massive dependent oedema had required drainage by needles placed into the subcutaneous tissues of the lower legs, and these puncture sites and accompanying stasis ulcers had become infected.

On 2 December, a young white woman, Denise Darvall, sustained a massive head injury after being hit by a car and was certified as having a lethal brain injury without any chance of recovery, by the neurosurgeon who had been called to treat the patient and who eventually referred her as an organ donor. There were no laws relating to brain death and organ transplantation in South Africa at that time, as elsewhere, and Barnard elected to take no chances. He invited the State’s forensic pathologist to the operating room, where ventilation of the donor (already prepared and draped for surgery) was discontinued. The blood pressure steadily fell and the heart arrested. The medical examiner pronounced that death had occurred. Barnard’s assistants then rapidly opened the chest, initiated pump-oxygenator support, cooled the heart to a low temperature and excised it.

The recipient had been prepared in the adjacent operating room and Barnard proceeded with the transplant. The enormity of what he was attempting was impressed upon him when, for the first time in his life, he looked into the chest and saw an empty pericardial cavity. The procedure went well and the heart functioned satisfactorily. No photographs were taken during the operation and so there is no visual record of this historic surgical procedure.

Within 48 hours the world’s press had descended on Cape Town and Barnard had become a household name. This intense public interest resulted in the appearance of Barnard and his transplant on the front covers of Time, Life, Newsweek and many other major foreign magazines within two to three weeks of the transplant. The world’s most publicised medical event had taken place.

Washkansky’s daily progress was followed intensely around the world, with almost every aspect of his care being made public. His early recovery was excellent, and the team was impressed with how rapidly the patient’s peripheral oedema was lost as his new heart functioned strongly. This excellent progress continued for almost two weeks, when Washkansky’s condition began to deteriorate and he developed radiographic infiltrates in the lungs. The surgical team was uncertain whether these were due to pulmonary oedema associated with cardiac failure from rejection, or with infection. Mistakenly, they initially elected to treat for rejection, intensifying the immunosuppressive therapy. This step was a lethal error as the patient had developed bilateral pneumonia, which was aggravated by the enhanced immunosuppression, and he unfortunately succumbed from severe pneumonia and septicaemia on the 18th day post-operatively.

## The second transplant

Not daunted by this failure, Barnard immediately selected his second patient, a 59-year-old local dental surgeon named Philip Blaiberg. The operation was performed on 2 January 1968. On this occasion, the surgical technique was slightly modified from the approach developed in dogs by Shumway and the Stanford group.^2^ The incision in the donor right atrium was extended from the inferior vena cava into the atrial appendage, thus avoiding the area of the sinus node.^3^ This modification has been utilised by nearly all surgical groups subsequently.

Blaiberg did well and was the first heart transplant patient to leave hospital. Media attention was enormous and his return to a relatively normal life was followed intensely over many months. It was Blaiberg’s success, perhaps more than any other single factor, that led to guarded optimism that heart transplantation would eventually prove a valuable treatment option. Blaiberg was the shining beacon, whereas the majority of other attempts at heart transplantation worldwide in the late 1960s and early 1970s seemed doomed to early failure. Blaiberg eventually died 19 months after his transplant. His autopsy demonstrated severe and widespread coronary artery disease. This was the first example of transplant coronary artery disease that now dominates as the major cause of graft failure after the first post-transplant year.^4^

The persistence of media coverage of heart transplantation at Groote Schuur gave Chris Barnard immense public and professional recognition on one hand, but on the other also evoked mixed and even heated reaction at times, regarding the question of ethics in organ transplantation. There were those critics who believed that this form of therapy was an unjustifiable and unethical form of palliation. Others responded with tremendous enthusiasm, as reflected by the fact that over one hundred heart transplants were performed in various centres all over world within the next year (1968).

However, the rather poor results following this experience, with a two-year survival of only 11%, had a negative effect on the initial enthusiasm over heart transplantation (Table 1). Only a few institutions maintained active heart transplant programmes, Cape Town being one of these. Barnard stated in 1970: ‘To curb transplantation at this stage would be to strangle one of the most promising and exciting fronts of medical endeavour of this century. From the experience gained in the problems of rejection, methods of immunological control will be improved and vital organ replacement will become a routine and life-saving procedure. To deny medicine its full thrust in this direction would be irresponsibly shortsighted. Indeed, it is difficult not to conclude that withdrawal from this new frontier would be professionally unethical. We have only to continue transplantation on a most active scale.’

**Table 1:** Worldwide Heart Transplant Results 1968–1970

*Year*	*Number of transplants*	*1-month survival*	*1-year survival*	*2-year survival*
1968	102	54	19	10
1969	48	28	7	6
1970	16	10	4	3
Total	166	92	30	19
Survival (%)		55	18	11

During the 1970s, Barnard’s programme, together with those at Stanford (under Norman Shumway), the Medical College of Virginia, and Hôpital La Pitie in Paris, were the only major centres continuously performing heart transplantation, and therefore the only centres where advances in heart transplantation were pioneered.

## Orthotopic heart transplantation

Between December 1967 and November 1974, exclusively orthotopic heart transplantation was performed. Ten such heart transplants were performed at Groote Schuur Hospital. The results, although poor by today’s standards, were exceptional when one considers the primitive nature of the immunosuppressive therapy available at the time (azathioprine, corticosteroids and antilymphocyte serum), and the team’s lack of experience in diagnosing and treating rejection episodes.

Of the 10 orthotopic heart transplants, four lived for more than 18 months, two of whom became long-term survivors. Dorothy Fisher lived for over 13 years and Dirk van Zyl lived for over 23 years.^5^ The remarkable fact about Dirk van Zyl is not only the longevity of survival but the excellent recovery from the operation. Within three months he returned to work and did not miss a single day’s work for the next 15 years, at which time he retired.

This early experience established sound criteria for the selection of recipients who would derive maximum benefit from heart transplantation. Experience was also obtained in methods of diagnosis and treatment of the acute rejection.

## The development of heterotopic heart transplantation

In 1973, Barnard performed a heart transplant and the donor heart failed to function satisfactorily, so the patient died in the operating theatre. When Barnard came out to break the sad news, he was asked why he could not put the old heart back, as at least it had kept the patient alive. This struck Barnard as a distinct possibility. If the patient’s own heart had been left in place, and the transplant was inserted as an auxiliary pump, failure of the donor heart may not have caused the patient’s demise. Furthermore, during severe rejection episodes, which were common in those early days and a major cause of the poor results at the time, the native heart might be able to maintain the circulation while rejection was reversed by increased therapy.

Barnard set Jacques Losman, a junior surgeon on his team, to develop the surgical technique of heterotopic heart transplantation in the animal laboratory. The concept was for a second heart to be placed in the right chest and for the two hearts to function in parallel.^6^ Two techniques were developed in the laboratory, in one of which the donor heart assisted the left ventricle only and another in which biventricular assist was provided. Only two left ventricular assist procedures were performed in patients, the remaining operations involving biventricular support.

## Heterotopic heart transplantation: the clinical programme

Forty-nine consecutive heterotopic heart transplants were performed in Cape Town between 1974 and 1983, with moderately good results for that era.^7^ Three of the first five patients survived more than 10 years. During this time it became clear that if irreversible rejection and failure of the donor heart developed, excision and replacement of the donor heart was not only technically difficult, but associated with significant morbidity. At the ‘re-transplant’ operation, it was preferable to replace the patient’s native heart by performing an orthotopic transplant, leaving the original heterotopic transplant *in situ*, even if it were no longer functioning. This prevented the necessity of dissecting the donor heart from the right lung, to which it might be tightly adherent.

Two 14-year-old boys, both of whom initially received heterotopic transplants, underwent a second (orthotopic) heart transplant for graft atherosclerosis, and were therefore the first patients in the world to have two donor hearts in their chest at the same time. The first of these remains well, 29 and 26 years after the hetero- and orthotopic transplants, respectively. In the other, the second transplant also eventually failed and he underwent a third transplant, again in the orthotopic site, and thus became one of the few humans to have had four hearts in his lifetime.

With the introduction of cyclosporine and the greatly reduced incidence of severe life-threatening rejection episodes, Barnard’s group resumed orthotopic heart transplantation. Heterotopic heart transplantation now has only a very small role, in, for example, the treatment of patients with fixed increased pulmonary vascular resistance, or when there is donor–recipient heart size mismatch.^8^

## Xenotransplantation

The ability of the heterotopic heart to provide temporary circulatory support to a failing native heart, in the hope that the native heart would recover, was extended into the realm of xenotransplantation. On two occasions in 1977, when a patient’s left ventricle failed acutely after routine open-heart surgery and when no human donor organ was available, Barnard transplanted an animal heart heterotopically. On the first occasion, a baboon heart was transplanted, but this failed to support the circulation sufficiently, the patient dying some six hours after transplantation. In the second patient, a chimpanzee heart successfully maintained life until irreversible rejection occurred four days later, the recipient’s native heart having failed to recover during this period.^9^ Further attempts at xenotransplantation were abandoned and even now, more than 30 years later, xenotransplantation remains an elusive holy grail despite decades of research.

## Hypothermic perfusion storage of the donor heart

Largely through the work of a young biochemist working in Barnard’s department, Winston Wicomb, a hypothermic perfusion system for storing hearts *ex vivo* for up to 48 hours was developed. A baboon’s heart could be stored by hypothermic perfusion and then replaced in the original baboon, which had been kept alive during this period by an orthotopic cardiac allograft. Some baboons were followed for up to two years after these procedures and showed normal cardiac function and myocardial histology throughout this period.^10^

With the success of this storage system in the laboratory, Barnard encouraged his team to use it in the clinical transplant programme. This would enable the transportation of hearts from distant centres in South Africa, which had hitherto been impossible, all donor hearts being procured locally in Cape Town. The device was used successfully on several occasions.^11^ However, in contrast to the experience in animals, it was commonly found that the human transplanted hearts had delayed graft function which often took many hours to recover. It was believed that this phenomenon of delayed function suggested temporary depletion of myocardial energy stores, related to the fact that, whereas in the baboon experiments the heart had been removed from a healthy anaesthetised animal, in the clinical situation the heart had been excised from a brain-dead subject.

## Investigations into the haemodynamic and metabolic effects of brain death

This alerted the team to the fact that brain death must have a detrimental effect on myocardial function, and led to extensive investigations into the haemodynamic and metabolic changes during and after brain death, and the implications of these changes. This work was the first comprehensive study investigating the effects of brain death.^12^-^14^ The major haemodynamic changes that take place as brain death was developing, some of which had been previously recorded by Harvey Cushing in 1902, were monitored.

It was documented that these could have a detrimental effect on subsequent myocardial function and were sometimes associated with histopathological features of myocardial injury. Previously unrecognised major endocrine changes that occur following brain death were documented. These include a massive catecholamine surge and a depletion of thyroid hormones and insulin, and other neuro-hormonal effects. These were associated with a loss of myocardial energy stores and other detrimental effects on cardiac function. Experimental studies in baboons indicated that aerobic metabolism soon ceases after brain death, and life is sustained by anaerobic metabolism.^15^

The concept of hormone-replacement therapy was proposed, particularly with regard to thyroid hormone replacement, which experimentally was reported to be beneficial to the maintenance and improvement of myocardial function. Subsequently, this therapy was administered to a number of human heart donors, with documented improvement in haemodynamic function.^16^ In recent years, hormonal therapy of the potential organ donor has gained increasing acceptance worldwide.^17^

## Brain death and ethical issues

As previously mentioned, there were no laws relating to brain death at the time of the first transplant. This undoubtedly helped to accelerate the growth of bioethics and to wrestle from the medical profession the monopoly over medical ethics, which they previously held. In February 1968, three months after the first heart transplant, a bill was introduced in the US Congress to ‘establish a commission to assess and report on the ethical, legal, social and political implications of medical advances’. It was evident that transplantation had raised serious ethical and legal questions for society.

Doctors were still very much committed to the view that the profession should make the decisions about contentious issues such as the moment of death and when to stop certain treatment. Most of the medical witnesses who appeared before the congressional hearings were opposed to the bill. Many did not resent input from philosophers or theologians but most felt that doctors ought to retain control and have the final say. None was more outspoken than Christiaan Barnard whose opposition to the proposal was ‘unqualified, almost nasty, perhaps reflecting the fact that in South Africa a doctor’s authority was still unchallenged or that he was not dependant on Congress or the National Institute of Health (NIH) for funding’.

The debates surrounding brain death and related ethical issues continued for many years and in 1981 the President’s commission in the United States declared that individual death depended on either irreversible cessation of circulatory and respiratory functions or irreversible cessation of all functions of the entire brain. In 1976 the UK Conference of Royal Medical Colleges accepted the view that damage to the brainstem was the crucial factor causing profound irreversible coma, and this became the definition of brain death.^18^

Barnard was also a champion of the disadvantaged and the poor, and an opponent of racism and apartheid, who welcomed its demise. He did his best to not allow racial segregation of patients within his department, in defiance of Government policy to segregate patients in hospitals according to race, as elsewhere in South Africa. Nevertheless nationalist politicians undoubtedly exploited him in the years following the first transplant, in order to improve the image of South Africa around the world, a time when repression was at its fiercest within the country and the worldwide condemnation of the apartheid regime was on the increase.

Raymond Hoffenberg, a colleague of Barnard’s and an exceptional academic doctor did not allow himself to be exploited by the Government and took a firm stand against the injustices which were perpetrated on its citizens in order to maintain apartheid; he was served with a banning order which forced him into voluntary exile a few weeks after Barnard’s surgical triumph. He went on to be a leading physician in Britain, eventually becoming president of the Royal College of Physicians and being knighted by the Queen.

## Christiaan Barnard in retirement

By the early 1980s, Barnard was tiring of the stresses and strains of clinical heart surgery and was losing interest in running a busy department. Furthermore, rheumatoid arthritis in several joints was causing him constant pain and making it increasingly difficult to operate to his satisfaction. In 1983, at the age of 61, he took early retirement from Groote Schuur Hospital and the University of Cape Town.

Barnard took the opportunity to pen an autobiography, *One Life*, which sold widely throughout the world.^19^ He generously donated the royalties to the Chris Barnard Fund which supported research into heart disease and organ transplantation at the University of Cape Town, and from which many subsequent researchers have benefitted significantly.

Soon after announcing his retirement, Barnard was invited to act as a consultant at Baptist Medical Center in Oklahoma City, where a new heart transplant programme was being planned. He spent six months a year for a number of years in Oklahoma, advising on the establishment of this programme, although not participating in the actual surgery. He finally retired from medicine in 1988 and settled back in Cape Town. He remained active as an international speaker on medical matters of general interest and continued to travel widely. He died during one of his travels on the island of Cyprus on 2 September 2001.

Chris Barnard’s department at Groote Schuur Hospital and the University of Cape Town has subsequently taken his name and has maintained an active heart transplant programme despite financial constraints imposed by the present Government’s reprioritisation of health expenditure on primary and preventative healthcare in a new post-apartheid South Africa, and the negative impact of HIV and AIDS on the healthcare sytem.^20^ Groote Schuur and associated academic hospitals are still the only public hospitals undertaking heart transplantation in Africa.

While no longer actively involved in basic laboratory transplant research, the Chris Barnard Division of Cardiothoracic Surgery at the University of Cape Town still has an internationally renowned cardiovascular research laboratory, directing its current research into methods of improving the surgical management of cardiac valvular and coronary diseases, which are more relevant to the cardiovascular diseases common in the African population. The most recent advance from this research unit has been the development of an external support for venous conduits used for coronary artery bypass grafting, which promises to increase the long-term patency of these grafts, and an international clinical trial using this mesh is currently underway.^21^
